# The Use of Sheep Movement Data to Inform Design and Interpretation of Slaughterhouse-Based Surveillance Activities

**DOI:** 10.3389/fvets.2020.00205

**Published:** 2020-04-24

**Authors:** Julie M. Stirling, Jude I. Eze, Geoffrey Foster, Aaron Reeves, George J. Gunn, Sue C. Tongue

**Affiliations:** ^1^Epidemiology Research Unit (Inverness), Department of Veterinary and Animal Science, Northern Faculty, Scotland's Rural College (SRUC), Scotland, United Kingdom; ^2^Biomathematics and Statistics Scotland, JCMB, Edinburgh, United Kingdom; ^3^SRUC Veterinary Services (Inverness), Northern Faculty, Scotland's Rural College (SRUC), Scotland, United Kingdom

**Keywords:** surveillance, sheep movements, ovine, slaughterhouse, sampling design

## Abstract

The design of surveillance strategies is often a compromise between science, feasibility, and available resources, especially when sampling is based at fixed locations, such as slaughter-houses. Advances in animal identification, movement recording and traceability should provide data that can facilitate the development, design and interpretation of surveillance activities. Here, for the first time since the introduction of electronic identification of sheep, the utility of a statutory sheep movement database to inform the design and interpretation of slaughter-house based surveillance activities has been investigated. Scottish sheep movement records for 2015–2018 were analyzed in combination with several other data sources. Patterns of off-farm movements of Scottish sheep to slaughter were described and the spatial distribution of several distinct slaughter populations, throughputs and catchment areas for Scottish slaughterhouses were determined. These were used to evaluate the coverage of a convenience-sample slaughter-house based survey for antimicrobial resistance (AMR). In addition, non-slaughter sheep movements within and between Scottish regions were described and inter-and intra-regional movement matrices were produced. There is potential at a number of levels for bias in spatially-associated factors for ovine surveillance activities based at Scottish slaughterhouses. The first is intrinsic because the slaughtered in Scotland population differs from the overall Scottish sheep slaughter population. Other levels will be survey-dependent and occur when the catchment area differs from the slaughtered in Scotland population and when the sampled sheep differ from the catchment area. These are both observed in the AMR survey. Furthermore, the Scottish non-slaughter sheep population is dynamic. Inter-regional movements vary seasonally, driven by the sheep calendar year, structure of the Scottish sheep industry and management practices. These sheep movement data provide a valuable resource for surveillance purposes, despite a number of challenges and limitations that were encountered. They can be used to identify and characterize the spatial origin of relevant populations and so inform the interpretation of existing slaughterhouse-based surveillance activities. They can be used to improve future design by exploring the feasibility and cost:benefit of alternative sampling strategies. Further development could also contribute to other surveillance activities, such as situational awareness and resource allocation, for the benefit of stakeholders.

## Introduction

Slaughterhouses provide a relatively easily accessible captive livestock population from which information on visible conditions can be obtained, or samples can be taken (1). Whether it is for ongoing, or targeted, surveillance or for other purposes associated with livestock health and welfare and disease control, the interpretation of any data that arises needs to be placed in the context of the population from which it has been, or is to be, obtained and that to which the resultant estimates may be extrapolated. The robustness of any resultant estimates are dependent on their reliability, validity, any bias (2), the epidemiology of the disease, condition, or case of concern (3), in addition to the questions asked as defined by the aims and objectives of the investigation.

The livestock populations that can be accessed at slaughterhouses are a natural subset of the general population. They are however a potentially biased subset due to their relative health and, in many cases, age at slaughter (4, 5). In addition, there can be spatial and temporal variations in the origins of the populations from which individual slaughterhouses obtain their throughput. When related to the epidemiology of the disease, condition, or case of concern, these variations can become relevant to design and interpretation of surveillance activities.

With legislative requirements and technological advances in the identification and recording of livestock, it should now be possible to provide improved quantitative evaluations of livestock population demographics, locations and movements. This information could then be used to inform both disease control strategies and the design of monitoring, data collection and surveillance activities. For the most part, published literature of analyses of animal movement data has focused on network analysis and risk-based surveillance, predominantly in the cattle and pig sectors [e.g., (6–11)]. Sheep sector movements have been included where they contribute to the spread of specified disease(s) under investigation (12, 13) or where a larger scale, multi-sector, perspective is taken (14). There are a few publications of sheep population demographics (15, 16) and network analyses (17, 18). In addition the spatial distribution of the 2002–2005 active surveillance sampling for the transmissible spongiform encephalopathies of sheep in Great Britain was explored using a “shot”-tracing method (19). There is, however, a dearth of literature relating to the use of livestock movement data in relation to design and interpretation of slaughterhouse-based activities.

During the period 2002–2007, unpublished work by the then Veterinary Laboratories Agency (now Animal and Plant Health Agency, APHA) explored the use of the British cattle traceability system data to investigate the relationship of source farms to slaughterhouses. These analyses were extended to the ovine population of England and Wales in order to inform the design of a potential slaughterhouse survey for prion protein genotype frequency in the lamb population. The outputs of the spatial analyses were combined with slaughterhouse throughput data, qualitative information, and expert opinion (20). At that time, identification of sheep was fairly crude at an individual animal level and electronic identification (EID) was not used; however, the use of geographical information systems (GIS) allowed exploratory descriptive analysis of the datasets. These analyses formed an integral part of understanding the target and sample populations and their inter-relationships; thus facilitating the design of potential sampling strategies.

Since then the use of EID for individual sheep identification and improved movement recording has been introduced via European law (21). In Scotland, rules for the identification and registration of sheep and goats have been implemented and are enforced through the Sheep and Goats (Records, Identification and Movement, Scotland Order, 2009, http://www.legislation.gov.uk/ssi/2009/414/made). Scottish sheep keepers are required to identify and register their animals. If the animals are to be moved off the holding, this movement must be recorded and reported to the Scottish Animal Movement Unit (SAMU). These movement data are held in a system known as the Scottish Livestock Electronic Identification and Traceability database (ScotEID).

The primary aim of this study was to assess the utility of the Scottish sheep movement data, currently collated and held in ScotEID, to determine whether they can be used to inform the design and interpretation of Scottish sheep slaughterhouse-based surveillance activities. A further objective was to apply this knowledge to an example, namely the 2017/18 sampling of sheep from one Scottish slaughterhouse for testing for AMR.

## Materials and Methods

### Data Sources and Description

All of the datasets used in this study were provided to the Scottish Government's Center of Expertise on Animal Disease Outbreaks (EPIC) by the agencies or institutions listed below and stored in the EPIC data repository; a centrally curated collection of data resources. This was established in 2011, to support research within EPIC as part of the Scottish Government's Strategic Research Programme.

#### Scottish **Sheep** Movements—ScotEID

ScotEID is the livestock traceability system for Scotland managed by the Scottish Agricultural Organization Society (SOAS) on behalf of the Scottish Government. The primary functions of the ScotEID systems are sheep, pig, and cattle movement recording. All sheep movements that contain all or part of their movement within Scotland are recorded in ScotEID.

Each movement record registers the departure premises, read location and destination premises. These are identified by their County Parish Holding number (CPH). A holding is a place where livestock are kept or handled in pursuit of an agricultural activity. In addition to farms, holdings include other types of premises and/or land such as markets, lairages, slaughterhouses, ports and showgrounds (https://www.gov.uk/guidance/register-land-you-use-to-keep-livestock).

For sheep, a movement record for a batch consists of the movement date, the number of animals moved and the number of reads. When a sheep with an electronic identification (EID) tag is scanned at a Critical Control Point (CCP), such as a market or slaughterhouse, there will also be a separate individual animal-level read record of their movement.

#### Scottish June Agricultural Census

The Scottish June Agricultural Census (Rural and Environmental Science and Analytical Services Division of the Scottish Government (RESAS) is sent annually to all holdings that complete a Single Application Form (i.e., which is for farmers who wish to claim payments under a number of support schemes) and to a random sample of other agricultural holdings. Information is collected across all types of farms about the areas owned or rented, crops, livestock numbers (not including cattle), and labor. Once every 10 years a full census is held with the last one being in 2012. It is a legal requirement to complete the census (Agriculture Act, 1947). For further information please see https://www2.gov.scot/Topics/Statistics/Browse/Agriculture-Fisheries/Publications/JuneAgriculturalCensus. The data provide a snapshot of sheep numbers during the same month (June) each year.

#### Other Data Sources

Premises types and geo-referenced locations were collated from a number of other Scottish and British (Great Britain, GB) sheep movement and demographic datasets using the unique CPH as the link between records. These data sources included: the Sheep and Goat Inventory (SGI, APHA) and the Animal Movement Licensing System (AMLS, APHA). These locational data included GIS coordinates and were combined to form a consistent repository of data. This was used to provide geo-references for the descriptive spatial analysis and to determine premises types.

### Data Analysis

The time period studied was the 4 year period 2015–2018, inclusive. Where illustrative single maps are provided they are for 2017.

Data processing and analysis were carried out using a combination of bespoke applications written in C++ and R (22) with packages, RPostgres (23), ggplot2 (24), pheatmap (25), and RStudio (26). Spatial analysis was performed using QGIS 2.14.8 (27).

#### Total Scottish Sheep Population

The Total Sheep Population in terms of number of sheep per calendar year was extracted from the Scottish Agricultural Census. The spatial distribution of the total sheep population was mapped as the sheep density calculated at a parish level as sheep per hectare (Shape file agricultural parishes_2016 from Scottish Government SpatialData.gov.scot).

#### Total Scottish Sheep Movements

Sheep movement data for the study period were extracted from the ScotEID database held in the EPIC data repository, reviewed and summarized. The number of movements and the number of sheep per movement in batch data were counted and compared with the individual animal-level data.

The data were split into four classes of movement types: the total movements, moves within Scotland, moves out of Scotland and moves into Scotland. The distribution of batch sizes in each class was visualized as a boxplot. The differences in the batch sizes between classes were compared using a generalized linear model (GLM) in R. Given that the batch sizes (number of animals in each batch) are counts, they were modeled as Poisson distributed. The batch sizes from different movement classes were considered to be significantly different from the reference category if the *p-*value of the odds ratio was <5%. Batch sizes for moves to slaughter (section Scottish Sheep Slaughter Populations) were also analyzed as above.

#### Scottish Sheep Slaughter Populations

Scottish holdings were defined by their CPH number and those that supplied sheep to any GB slaughterhouse were identified from the movement records. A sheep movement record was classified as contributing to the slaughter population when the destination location was any GB slaughterhouse.

Batch movement data were joined to individual animal movement data through common variables and the resulting dataset used to determine the numbers of sheep received by each slaughterhouse. From batch data it can be determined how many animals moved to a slaughterhouse on a particular date. However, when the datasets were combined it was made possible to identify some animals that had their EID tags read at both the market and the slaughterhouse. In the batch data these were recorded as two separate movement records, thus effectively double-counting some of the sheep moving to a slaughterhouse e.g., Record 1: Holding = departure premises; market = read location; slaughterhouse = destination. Record 2: market = departure premises; slaughterhouse = read location; slaughterhouse = destination premises. By removing the second move from the analysis this reduced the chance of over-estimating the number of sheep going to slaughter.

The number of batch movements and the numbers of sheep to each of the slaughterhouses were calculated for each year. The slaughterhouses were ranked and the annual throughput determined.

Data were processed to a consistent form for each of the 4 years (2015–2018) and three major populations were described.

- **Population 1** - The total annual **Scottish Sheep Slaughter Population** (SSSP) contains sheep originating from a Scottish holding (hereafter referred to as Scottish sheep) that have been identified as going directly to slaughter at any slaughterhouse throughout Great Britain (GB). This Population was made up of two subsets, namely:
- **Population 2** - The **Scottish Sheep Slaughtered in Scotland Population** (SISP) contains Scottish sheep that have been identified as going directly to slaughter at a Scottish slaughterhouse.
- **Population 3** - The **Scottish Sheep Slaughtered outside Scotland Population** (SOSP) contains Scottish sheep that have been identified as going directly to slaughter to a slaughterhouse outside of Scotland.

There is one further minor group, Population 4 which consists of sheep supplied in a direct move for slaughter at some Scottish slaughterhouses (i.e., they constitute a proportion of the throughput of those slaughterhouses) that originate from holdings out with Scotland. These sheep are part of the population of sheep slaughtered in Scotland but did not come from a Scottish farm so were excluded from this analysis.

#### Descriptive Spatial Analysis

Precise co-ordinates were used to plot holding locations, if they were available; if not, the parish centroid was used. Slaughterhouses and their direct supplying premises were plotted on the GB national grid with QGIS 2.14.8. A grid consisting of 115 km^2^ hexagons was created using the QGIS Processing plugin module, overlaid on the point data and trimmed to the coastline. The number of holdings in each hexagonal grid cell or the number of sheep that were supplied to slaughter from each grid cell were counted and density maps plotted using these calculations.

Slaughterhouse catchment areas are defined as the spatial distribution of the holdings from which they receive their annual throughput.

#### An Exemplar—The Antimicrobial Resistance Survey 2017/18

From June 2017 to March 2018 pilot sampling at slaughterhouses was undertaken by Food Standards Scotland (FSS) to test for antimicrobial sensitivity testing (AST) by SRUC Veterinary Services (VS). The aim was to acquire 40 samples per species per month from cattle, sheep, pigs and poultry, with each species being sampled in a single week of each month, in rotation. For sheep, the sampling was done at a single slaughterhouse (Slaughterhouse A). For cost and logistical reasons this was a convenience sample with the sampling occurring on 1 day in the designated week, over a 4.5 h period. The sampler was instructed to try to obtain samples from a spread of farms. Individual fecal samples were obtained. These were sent with a copy of the accompanying Food Chain Information (FCI) form to SRUC VS (Inverness) for AST, by disc diffusion of one *E. coli* isolate per sample against an agreed panel of twelve active substances (28). European Committee on Antimicrobial Sensitivity Testing (EUCAST) methodology and clinical breakpoint interpretative criteria were followed.

The ovine dataset consists of 388 records. Each record represents a single sheep fecal sample collected between June 2017 and March 2018. The samples are identified by a sample reference number, an Animal flock ID based on the eartag number (UKxxxxxx) and the holding CPH.

The spatial distribution of the sampled holdings was compared to the distribution of holdings from the slaughterhouse catchment area. In order to establish the representativeness of the sampled holdings across the slaughterhouse catchment areas, the observed number of sampled holdings was compared to the number of holdings that would be expected if sampling was allocated proportionately based on spatial spread or clustering of holdings. This was achieved by determining which hexagons were adequately represented and which were over represented by the sampled holdings using chi square test. Considering that the data is large (chi square degrees of freedom = 213) and given that the Chi square is asymptotically normally distributed when the sample size is large, the cut-off point for significance was taken to be 2. Therefore, if the calculated Chi square is ≤ 2 the hexagon is deemed to be adequately sampled otherwise the hexagon is deemed to be oversampled.

#### Non-slaughter Sheep Movements Within Scotland

Sheep movement data extracts were generated and processed. A shapefile (Supplementary Figure 2) of the Scottish Agricultural Regions was created based on https://www2.gov.scot/Topics/Statistics/Browse/Agriculture-Fisheries/AgricMapRegions. Sheep holdings were plotted using GIS coordinates and the CPH numbers identified that fell within a particular region. The number of sheep moving into or out of each Scottish region was calculated for 2017 as a whole and as numbers per quarter. This analysis was restricted to sheep movements that had originated and terminated within Scotland only, with movements to slaughter omitted. Movement matrices, or “heatmaps,” were then generated from this data. Sheep numbers were plotted as quartiles of the observed distribution of sheep numbers.

The movement matrix represents sheep movements between 14 regions. The numbers of sheep moving between regions were plotted either as (i) the number of sheep moving from departure region “X” to destination “Y” as a percentage of the number of sheep moved from departure “X” to all 14 destinations or (ii) the number of sheep moving to destination “X” from departure “Y” as a percentage of the number of sheep moved to destination “X” from all 14 departure regions. Sheep numbers were plotted as quartiles of the observed distribution of sheep numbers.

#### Seasonal Movements

In order to confirm seasonal patterns of sheep movements, the number of sheep moving each month was counted and compared for each year. These counts included the total number of sheep moved throughout the year or just the movements to slaughter. The differences in where a slaughterhouse sources its throughput from at different times of the year was analyzed by counting the number of sheep supplied to the slaughterhouse for each quarter of the year (January–March, April–June, July–September, and October–December). The number supplied from each grid cell was plotted on a hexagonal grid map as a percentage of the annual slaughterhouse throughput.

## Results

### Total Scottish Sheep Population

**Figure 1:**
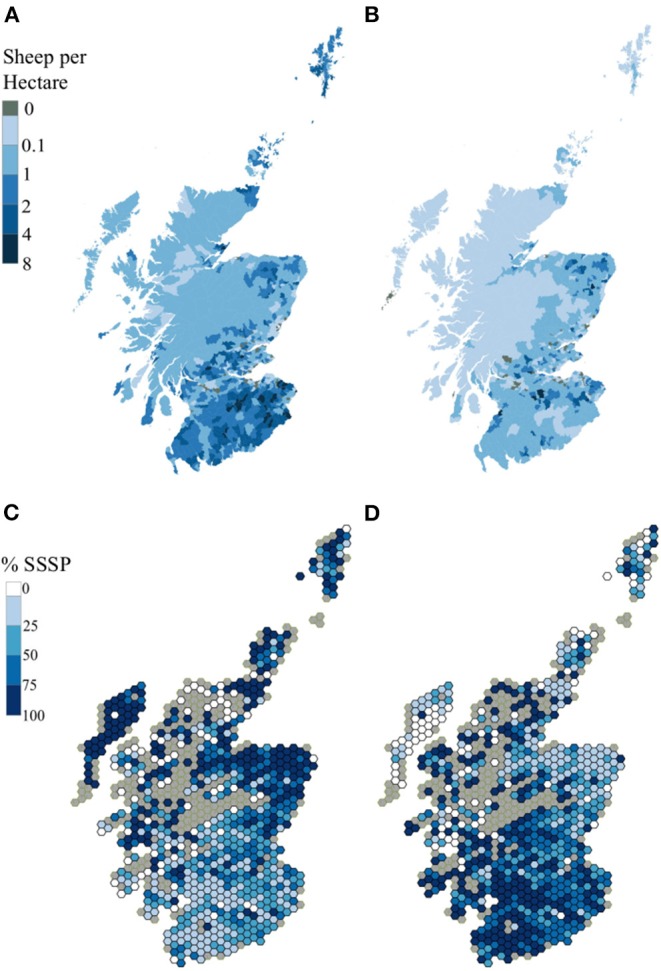
**(A–D)** Spatial Distribution of Sheep Populations using 2017 as the example year. **(A)** Total Scottish sheep population and **(B)** Scottish Slaughter Sheep Population (SSSP), both as sheep/hectare per parish in categories by quintile. **(C)** Scottish Sheep Slaughtered in Scotland (SISP) and **(D)** Scottish Sheep Slaughtered outside Scotland Population (S0SP) both as Percentage of the SSSP per 115 km^2^ hexagon.

Overall numbers of sheep in the Scottish June Agricultural Census sheep increased by 4% between 2015 and 2017 when numbers peak at just under 7 million (Table 1). Sheep are unevenly distributed throughout Scotland with the densities of sheep/hectare within parish varying from 0 in a few urban areas to a maximum of 8 (Figure 1A). Higher densities of sheep are concentrated in the southern part of the country (Borders, Clyde Valley, and Dumfries and Galloway) and to a lesser extent on the east coast (Highlands, Grampian, and Tayside) and Shetland Islands (Figure 1A).

**Table 1:** Overall annual summary of the Scottish sheep population, numbers of premises involved in any sheep movement in Scotland and the numbers of those sheep movements by batch, sheep and destination and as a % of total for 2015–2018, inclusive.

**Annual sheep movements**	**2015**	**2016**	**2017**	**2018**	**Average**
Scottish sheep population*	6,701,376	6,826,116	6,985,157	6,593,410	6,776,515
Premises (moving sheep)					
Total number	15,107	15,906	15,509	15,846	15,592
Number within Scotland	12,243 (81.0%)	12,587 (79.1%)	12,086 (77.9%)	12,348 (77.9%)	12,316 (79.0%)
Batch movements					
Total number	276,376	299,151	287,956	290,501	288,496
Number within Scotland	149,425 (54.1%)	149,665 (50.0%)	135,475 (47.0%)	139,220 (47.9%)	143,446 (49.7%)
Number out of Scotland	121,115 (43.8%)	143,015 (47.8%)	146,315 (50.8%)	144,389 (49.7%)	138,709 (48.1%)
Number into Scotland	5,836 (2.1%)	6,471 (2.2%)	6,166 (4.6%)	6,892 (5.0%)	6,341 (2.2%)
Sheep numbers moved					
Total number	4,533,375	4,690,748	4,547,346	4,285,308	4,514,194
Number within Scotland	2,319,498 (51.2%)	2,288,310 (48.8%)	2,085,289 (45.9%)	2,054,108 (47.9%)	2,186,801 (48.4%)
Number out of Scotland	1,925,597 (42.4%)	2,081,527 (44.4%)	2,142,856 (47.1%)	1,910,232 (44.6%)	2,015,053 (44.6%)
Number into Scotland	276,714 (6.1%)	312,217 (6.7%)	307,838 (6.8%)	310,115 (7.2%)	301,721 (6.7%)

The star * was used to indicate that the Scottish sheep population numbers come from the Agricultural census, while the movements come from ScotEID.

### Total Scottish Sheep Movements

When the number of sheep movements calculated from the batch records and from the individual read animal-level records were compared, there were, on average, 20% more sheep and 4.8% more batches recorded in the batch data for each year studied. There was an average of 15% of sheep found in batch data that did not have associated individual animal-level reads and an average of 4% individual animals were missing due to mis-reads.

Over a third (35%) of holdings in the sheep movement data did not have any spatial data associated with the record. Depending on the year, the total number of premises moving sheep to and/or from Scotland varied between 15,107 and 15,906; the majority of premises involved in these movements were located in Scotland (average across 4 years = 79%, Table 1).

A mean of 288,496 batches moved per year, of which half (49.7 %) moved entirely within Scotland. On average just under half (48.1%) originated in Scotland and moved out across the border, with only a small percentage of batches (2.2%) entering Scotland. However, this latter percentage more than doubled in 2017 and 2018, compared to 2015 and 2016 (Table 1).

In terms of individual animals, an average of 4,514,194 sheep moved annually, with the largest percentage (48.5%) moving entirely within Scotland. However, the percentage that moved into Scotland was greater than when considered as batches; with an average of 6.7% (Table 1).

The range of batch sizes making up the sheep movements during 2015–2018 was wide and skewed (Supplementary Figure 1). The mean batch size for all movements both within and out of Scotland was similar. However, that of movements into Scotland was significantly larger (*p* < 0.0001) when compared with movements within and out of Scotland (Table 2). The mean odds of total movements into Scotland were 3.7 times larger than those of the other movement classes. Similarly, the slaughter batch sizes moving into Scotland have odds that were 4.2 times larger than the slaughter batches moving within Scotland and 8.3 times larger than those moving out of Scotland.

**Table 2:** Summary statistics of batch sizes for Scottish Sheep movements 2015–2018.

**Batch movements**	**Batch sizes**				**GLM**
	**Min**	**Max**	**Mean**	**Median**	**Odds ratio**
**Total annual movements**					
All movements	1	1,410	16	5	0.27*
Movements within Scotland	1	779	15	4.75	0.27*
Movements out of Scotland	1	1,410	15	5	0.26*
Movements into Scotland	1	849	56	10.75	1
**Movements to Slaughter**					
All movements	1	1,073	16	5.5	0.16*
Movements within Scotland	1	563	24	7.75	0.24*
Movements out of Scotland	1	1,073	13	5	0.12*
Movements into Scotland	1	824	107	63.5	1

* *p < 0.0001*.

**Figure 2:**
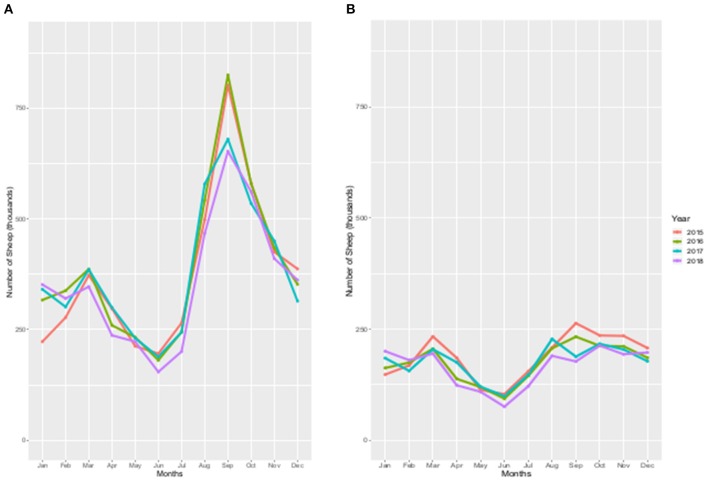
**(A,B)** The number of sheep moved per calendar month for each year 2015 to 2018. **(A)** Total sheep population, **(B)** Sheep to slaughter (SSSP).

There was seasonal variation in the number of sheep moved with a large peak in September (Figure 2A). There was a smaller peak in the first quarter of the year, usually in March and the lowest numbers of sheep were moved in June. The pattern was relatively consistent year on year, with some changes in magnitude between years. Over the study period the number of movements making up the autumn peak has reduced by 19%, relative to 2015, while the spring peak has remained fairly constant throughout 2015–2017. However, this spring peak decreased by 10% in 2018, when numbers of movements in March were on a par with those in January 2018 (Figure 2).

### Scottish Sheep Slaughter Populations

Approximately a third of the Scottish sheep population went to slaughter in each year (SSSP, Table 3) of the study period, through (on average) 63 British slaughterhouses. Over a third of these slaughterhouses (*n* = 22, 35%) were located in Scotland. Although the number of slaughterhouses slaughtering sheep of Scottish origin does not change markedly there were some differences with regard to which of the slaughterhouses were operable in each year.

**Table 3:** Numbers and percentages of sheep in the Scottish Sheep Slaughter populations 2015–2018.

**Scottish Sheep Slaughter Populations**	**2015**	**2016**	**2017**	**2018**
**Scottish Sheep Slaughter Population (SSSP)**				
Sheep numbers	2,254,262	2,090,816	2,111,158	1,973,890
Percentage of total Scottish sheep population	33.6%	30.6%	30.2%	29.9%
**Slaughtered in Scotland (SIS)**				
Sheep numbers	1,107,763	1,048,724	987,496	963,477
Percentage of SSSP	49.1%	50.2%	46.8%	48.8%
**Slaughtered outside Scotland (SOS)**				
Sheep numbers	1,146,499	1,042,092	1,123,662	1,010,413
Percentage of SSSP	50.1%	49.8%	53.2%	51.2%

An average of 8,543 distinct Scottish premises per year supplied sheep to slaughterhouses in GB. A quarter of these premises (25%) supplied Scottish slaughterhouses only; approximately a quarter (26%) supplied non-Scottish slaughterhouses only, while the reminder—just under a half (49%)—supplied sheep to slaughterhouses in both Scotland and the rest of GB. Approximately half of the Scottish sheep slaughtered in a year (SSSP) are slaughtered within Scotland (SISP, Table 3).

The lowest number of sheep moves to slaughter occurred in the period May to June of each year (Figure 2B). There was an annual March peak. Later in the year, between August and November, the peak in the number of sheep slaughter moves was flatter (Figure 2B) than that of the total sheep moves (Figure 2A).

### Descriptive Spatial Analysis

#### The Scottish Slaughter Populations

**Figure 3:**
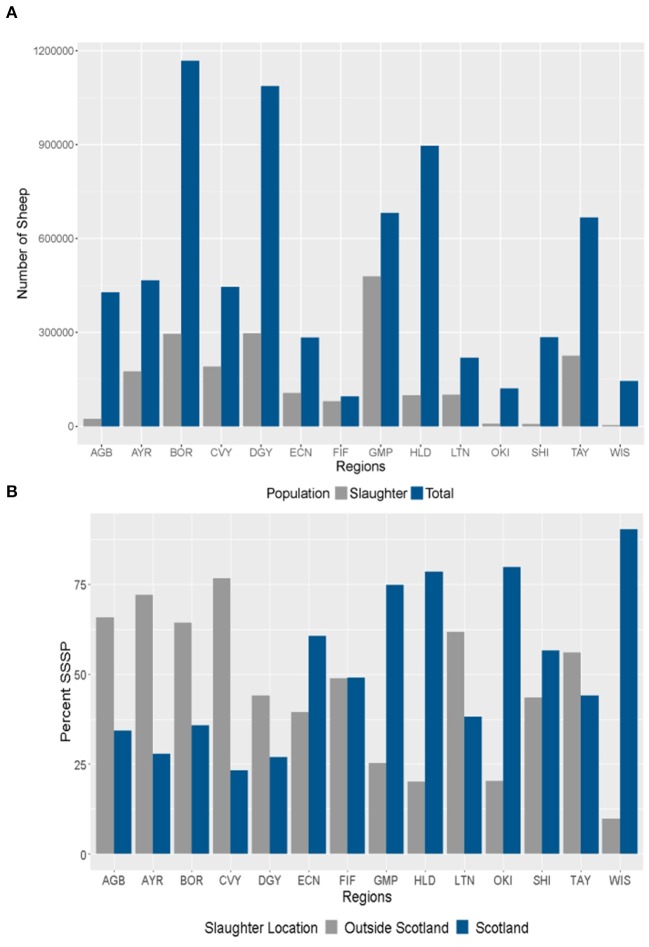
**(A,B)** Regional Distribution of Scottish sheep populations. **(A)** Numbers of the total sheep population (June Agricultural Census 2017) and Scottish Sheep Slaughter Population (SSSP) 2017 by region; **(B)** Percentage of the SSSP that were slaughtered in Scotland (SISP) and out-with Scotland (SOSP) by region.

Although broadly similar, the distribution of the Scottish sheep slaughter population (SSSP, Figure 1B) differed from that of the total sheep population (Figure 1A). It was generally more diffuse. There were specific foci of higher densities scattered throughout the north east, central and southern areas. The largest shares of the SSSP originated immediately from Grampian (22.9%), Dumfries and Galloway (14.2%) and the Scottish Borders (14.1%, Figure 3A). Over the 4 years of the study period 2015–2018, there did not appear to have been major changes in the geographical distribution of the Scottish sheep slaughter population (data not shown). For Region codes and location see Supplementary Figure 2.

The immediate origins of the SISP sheep that originated in Scotland and were sent to slaughter at a Scottish slaughterhouse—was not uniformly distributed across Scotland (Figure 1C). In each year, a greater percentage of SSSP sheep from the Highlands, Orkney, and Shetland Islands, the Western Isles, Grampian and East Central areas were slaughtered in Scotland than elsewhere in GB. For most of the central and southern regions the reverse is true (Figures 1C,D, 3B).

#### Slaughterhouse Rankings

**Figure 4:**
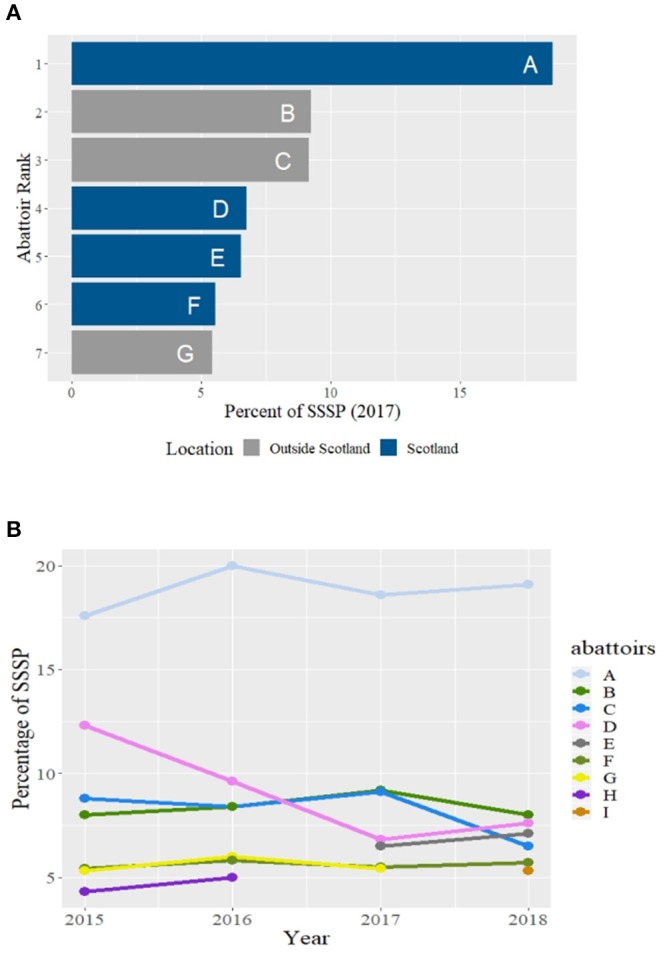
**(A,B)** Slaughterhouse ranking by throughput of numbers of the Scottish Sheep Slaughter Population (SSSP) per annum **(A)** Slaughterhouses with more than 5% in 2017 and their location. **(B)** Temporal ranking over the study period of Slaughterhouses with more than 5% throughput.

Ranked by throughput of SSSP, in terms of sheep numbers per annum, the same four slaughterhouses topped the ranking throughout the study period (2017 shown in Figure 4A). Each year seven British slaughterhouses each received more than 5% of the SSSP and between them they processed approximately two thirds of this population (Figure 4B). Although the top ranked slaughterhouse (A) remained the same, with a mean throughput of 19% of the SSSP per annum, the other six slaughterhouses changed slightly, as did their rank (Figure 4B). Four of these top-ranked slaughterhouses were located in Scotland (A, D, E, and F). They received more than a third (37.5%), in terms of sheep numbers, of the SSSP (Figure 4A).

If the throughput of the SSSP was estimated in terms of the number of batch movements rather than numbers of sheep, the ranking of British slaughterhouses that received sheep from the SSSP differed The majority of the top ranked premises are outside Scotland (data not shown).

**Figure 5:**
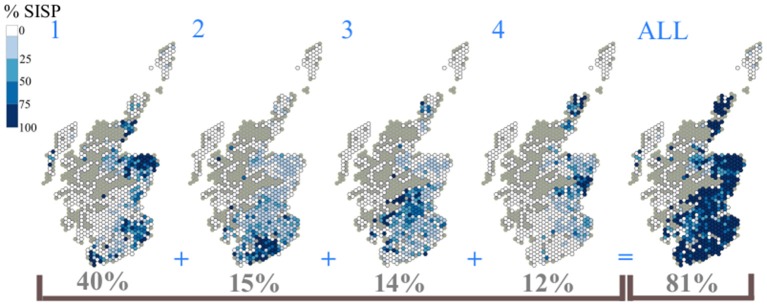
The catchment areas of the top four slaughterhouses in Scotland ranked by throughput in sheep numbers as a percentage of Scottish slaughter sheep, slaughtered in Scotland (SISP) in 2017.

The top four Scottish slaughterhouses (A, D, E, F) together received just over four-fifths (81%) of the population of Scottish slaughter sheep (SSSP) that were slaughtered within Scotland (SISP); 2017 (Figure 5).

#### Slaughterhouse Catchment Areas

Each Scottish slaughterhouse catchment area was different. There was some degree of overlap between some slaughterhouses and there were some slight variations in the distribution of the supply population over the study period. However, the catchment areas generally remain relatively discrete and stable over this 4 year period. The catchment areas of the top four ranked Scottish slaughterhouses supply 40, 15, 14, and 12% of the SISP respectively. In combination the resultant area gave substantial coverage of the mainland spatial distribution of the SISP (compare Figure 5 with Figure 1C). Some of the smaller slaughterhouses in terms of throughput had very localized catchment areas, especially those situated on the west coast, Western Isles, Orkney and the Shetland Islands, (plots not shown).

##### Seasonality of catchment areas

**Figure 6:**
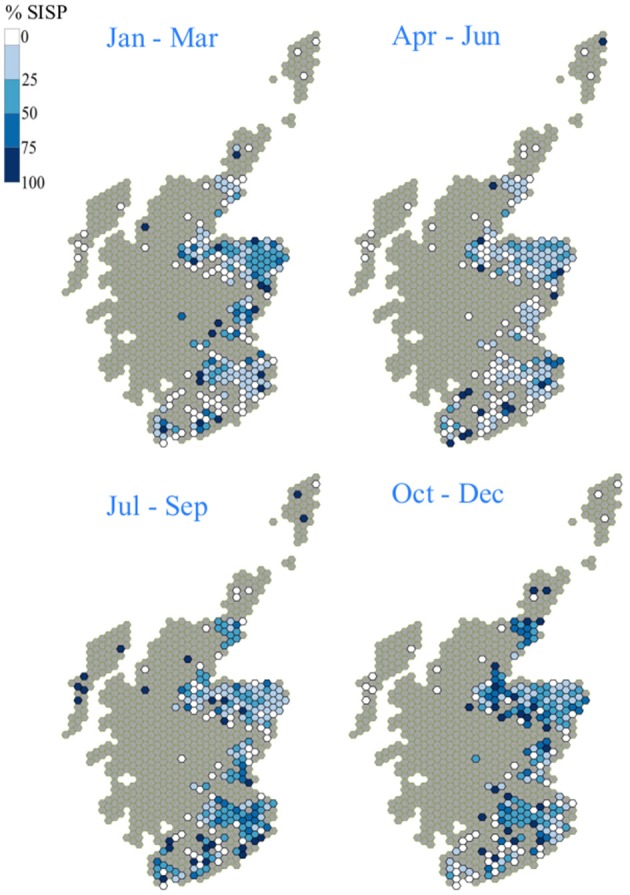
Spatial distribution of the source of the Slaughterhouse A's annual throughput by calendar quarter for 2017 as (% of the total throughput from a hexagon, January–March; April–June; July–September; October—December).

There were seasonal differences in the spatial distribution of premises supplying sheep to slaughter. For example: the movement of all of the sheep supplied from the Western Isles to slaughterhouse A occurred in the third quarter (July to September), whilst sheep from North East Highland (Caithness area) and Orkney tended to move to slaughter in the 4th quarter (October to December, Figure 6).

### An Exemplar—The Antimicrobial Resistance Survey 2017/18

**Figure 7:**
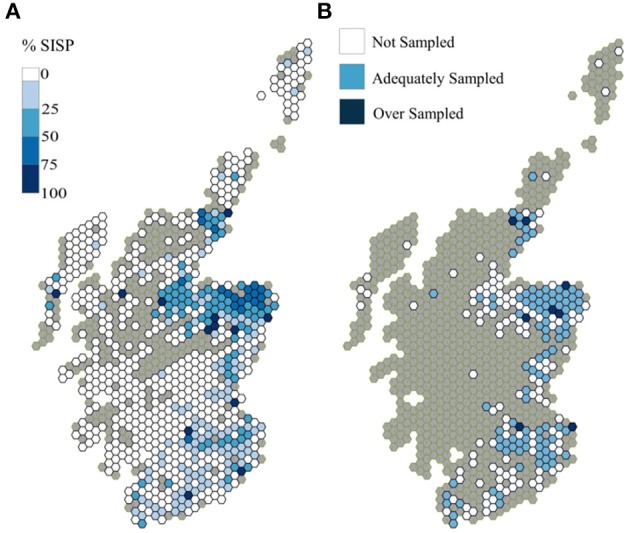
Distribution of Slaughterhouse A's throughput during the AMR survey period, on a holding basis **(A)** as a % of the holdings that supplied sheep to the Slaughtered in Scotland Population (SISP) in that period **(B)** degree of sampling achieved.

The slaughterhouse used for the AMR Survey (Slaughterhouse A) was the Scottish slaughterhouse with the highest throughput of sheep. Over the 4 year period of the sheep movements analysis, it averaged 19% of the total Scottish sheep slaughtered annually and 38% of those sheep slaughtered in Scotland (SISP), in terms of sheep numbers. The catchment area covered north-eastern Scotland and included some of the Borders and eastern Central Belt (Figure 7A). This slaughterhouse did not receive, or received few SISP sheep from the West Coast, Highlands, the Islands (Shetland, Orkney and Western Isles), the west Borders (Dumfriesshire, Clyde Valley), and central areas (Perthshire and Fife). A total of 7,771 distinct holdings throughout Scotland contributed to the SISP during the AMR study period, of which 1,023 were in the catchment area of slaughterhouse A.

Based on the CPH of the 388 fecal sample records, these samples came from 216 distinct holdings. Two samples had no CPH identifier. There were multiple samples from some holdings (Min = 1, Q1 = 1, Median = 2, Mean = 2, Q3 = 3, Max = 6). Two holdings, each supplying one sample, were omitted from the analysis because they were located outside Scotland (Northumberland).

When the spatial distribution of the sampled holdings was compared to the distribution of holdings from the slaughterhouse catchment area, 52% of the grid cells in the catchment area were deemed to be adequately sampled, 45% were not sampled and the remaining 3% were oversampled (Figure 7B).

The parts of the catchment area that were not sampled included the Shetland Islands and Western Isles, the western part of the northwest part of the northeastern area (around Inverness), the central east coast area (Angus and Fife) and the Central Borders area (Figure 7B). The catchment area already had low coverage of the SISP sheep, in all of these areas except the northwest part of the northeastern area (Figure 7A). As was seen earlier in the results, the SISP was not, in itself, spatially representative of the Scottish slaughter sheep population, due to the distribution of the “escaped” population, i.e., those that go to be slaughtered out-with Scotland (SOSP, Figures 1C,D).

### Non-slaughter Sheep Movements Within Scotland

**Figure 8:**
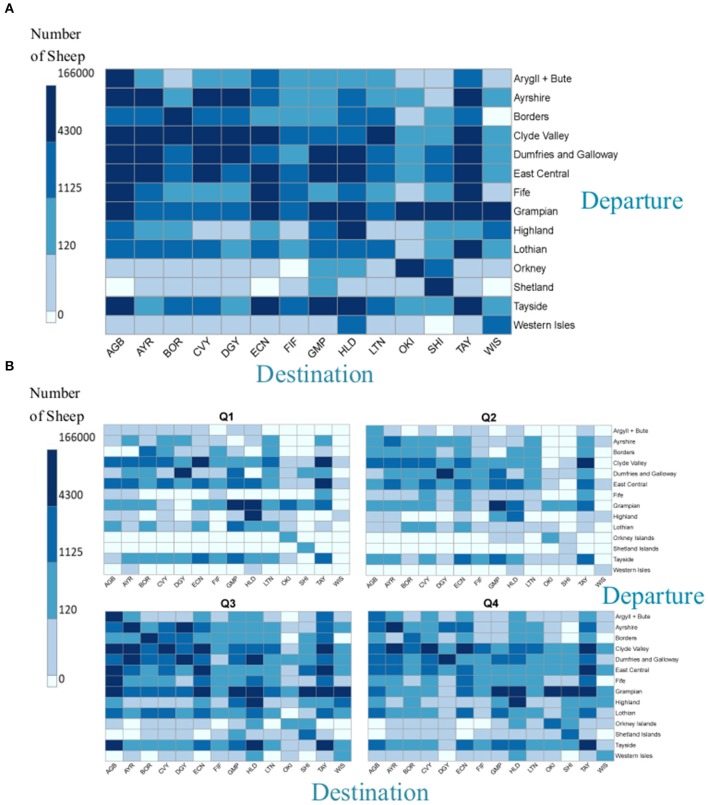
The numbers of sheep moved in non-slaughter related Scottish moves during 2017 **(A)** from departure region to destination region in 2017 **(B)** from departure region to destination region in each quarter of 2017 (January–March; April–June; July–September, October–December).

**Figure 9:**
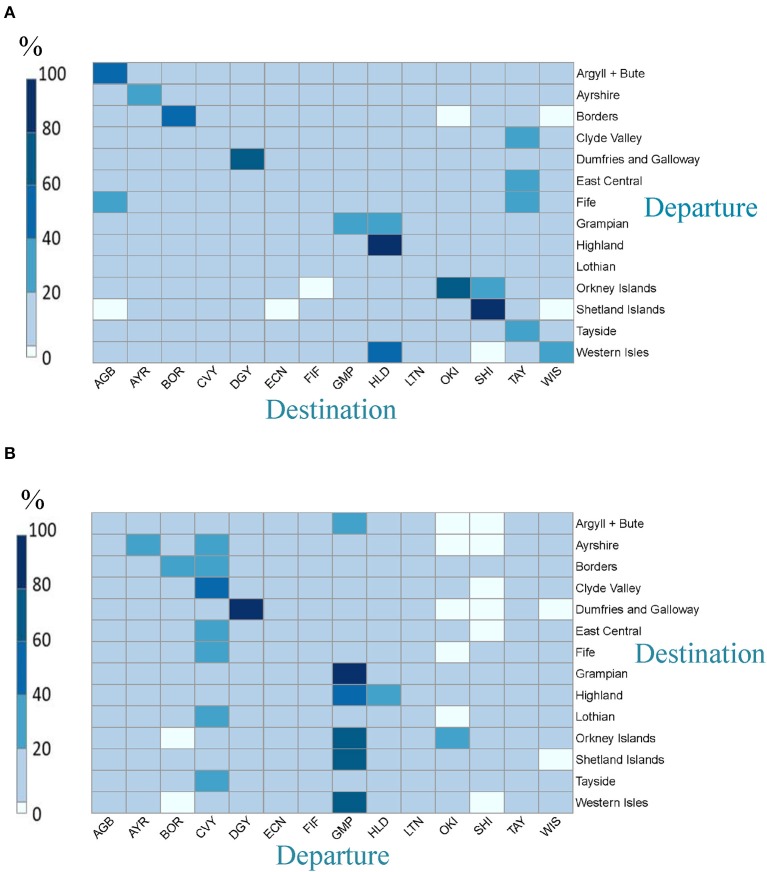
The numbers of sheep moved during 2017 **(A)** to a region (destination) as a % of total sheep moved from a region (departure) **(B)** from a region (departure) as a % of total sheep moved to a region (destination) i.e., horizontal line = 100%.

Excluding moves to slaughter, it was estimated that over a million (1,119,542) sheep were moved within Scotland in 2017. In general, the volume of sheep movement off Scottish holdings was much higher than the on-movement. The largest number of sheep were recorded as moving from premises within three regions; Grampian, Dumfries and Galloway and Clyde Valley (34, 20, and 13% of total sheep moved, respectively during the year, Figure 8A). Internal movements within each of these regions accounted for 38, 76, and 16%, respectively, of the number of sheep that departed from a holding within that region (Figure 9A). For most regions high numbers of sheep moved internally within the region over the year (the squares on the diagonal from top left to bottom right, Figure 8A). However, more sheep movements occurred from Lothian to Tayside than within Lothian. A large number of sheep in the Western Isles moved either within the Western Isles or to Highland (Figure 8A), with the highest percentage moving to Highland (Figure 9A).

Regions that were the destination for the highest number of sheep in 2017 were Highland plus Dumfries and Galloway, each with 17% of the total numbers of sheep moved; Grampian was the third most popular destination with 16% (Figure 8A). Many of these were from internal moves within the region: just over a quarter of moves (26%) with a Highland destination, came from within Highland (Figure 9B). This was lower than in Dumfries and Galloway and Grampian, where internal moves account for 89 and 81%, respectively.

#### Seasonality of Non-slaughter Sheep Moves Within Scotland

There was a seasonal difference in the numbers of non-slaughter sheep moved within Scotland (Figure 8B; January–March = 15%, April–June = 11%, July–September = 47% and October-December = 27% of the total number of sheep moved throughout 2017). However, there was also a seasonal difference between how the sheep moved between regions (Supplementary Figures 3A,B), two examples of which are described below.

Example 1: in the first half of the year (January–March and April–June) Grampian and Dumfries and Galloway sent and received most sheep overall. In the third and fourth quarter, while they still sent the most sheep, Highland became the region receiving most sheep from other Scottish regions. Example 2: in the first quarter, sheep from the Shetland Islands only moved within the Shetland Islands and a small proportion of sheep moved into the Shetland Islands from elsewhere. However, in the second half of the year, although sheep from the Shetland Islands still moved predominantly within Shetland, they also moved out to other destinations. These were Grampian in both the third and fourth quarters, Tayside in the third and the Orkney Islands in the fourth quarter (Supplementary Figures 3A,B).

## Discussion

In this study, for what the authors believe is the first time since the introduction of EID, the utility of national sheep movement data has been assessed to determine whether they can inform the design and interpretation of slaughterhouse-based surveillance activities. The conclusion that can be drawn from these analyses is that these data can be utilized in such a way, as illustrated by application of the analytical techniques developed during the assessment to an example: a survey for antimicrobial resistance in Scottish slaughter sheep.

A primary challenge to the development of all operational animal health surveillance systems is the availability of and access to existing data. This is often related to issues of subject confidentiality. In this study, this has been addressed by the Scottish Government's Center of Expertise on Animal Disease Outbreaks (EPIC) data repository. From its inception in 2011, a centrally curated collection of data resources has been carefully established, extended, improved and maintained. Data are sourced from a number of data providers, both public and private and held in a secure data repository. User access requires legally binding data use agreements to be in place and is monitored and audited. The existence of this repository facilitated the study, as multiple data sources were required to address some of the challenges identified.

A number of data limitations were encountered during the course of the study. These were: 1/ that there is no year-round centrally available record of individual sheep and their geo-location, until a movement occurs between premises and the EID is captured when read at a Critical Control Point; 2/ knowledge of the source and destination premises type and geo-location is compromised by missing data; 3/ accurate counting and identification of individual sheep within movements is difficult, even with EID; 4/ the age of the sheep moved is unknown, and 5/ only the origin from the move immediately prior to slaughter could be accurately determined. Some of these are inherent aspects of the technologies in use and data collection processes. Some are due to missing data, or gaps in the information available. As discussed below, these challenges limit the utility of these data both for this surveillance purpose, and for others, to varying degrees. Their existence highlights the need to take adequate care; both when using these data for analyses and when interpreting the outputs of any such analyses.

The first challenge is the denominator i.e., where individual sheep are at varying time-points throughout the year. Total sheep numbers by locations are only available twice in a year: in June from the Agricultural Census (RESAS) and in December from the SGI (https://www2.gov.scot/Topics/Statistics/Browse/Agriculture-Fisheries/Publications/SGAI-DAS). These provide snapshots of what is known to be a dynamic population. Numbers and the age-structure of sheep populations on holdings will vary between the two as the Census in June occurs after the peak lambing period (March-May). In December's Inventory, the majority of fat lambs will have gone to slaughter (29), store lambs will have moved on, replacement breeding stock will have joined their flocks and those intended as future breeding stock may have moved to winter quarters “on tack.” The spatial distribution will also be different from June. While the Census data are the best denominator for the pig sector (5, 11) and cattle movement data are now used to provide the numbers of cattle for the June Census, for sheep the SGI is more usually used (13, 30). In this study, the gross analysis of within-country non-slaughter movements demonstrated this spatially dynamic nature of the Scottish sheep population and provided context for discussion with regard to the fifth challenge (direct move only). It is reassuring that the outputs from this analysis, and the overall sheep numbers and movements, correspond to known aspects of the sheep calendar year, management activities and events. For example, the low level of within Scotland non-slaughter moves in the second quarter corresponds to the lambing and post-lambing season, while the decline in the spring peak in March in 2018, and the subsequent population fall at the June 2018 Agricultural Census are indicative of the extreme, adverse, weather conditions (known as “the Beast from the East”) during March 2018 when sheep couldn't move and a substantial number died (31).

When sheep do move, the second challenge arises. In this study, through access to and use of a number of other data sources, this was minimized. While technically a slaughterhouse should be identifiable by only having inwards animal movements, which was the case in these data, this is not a given (16). Centrally maintained, up-to-date, complete lists of the classifications of premises type for active non-farm (i.e., non-livestock producing premises—slaughterhouses, markets, collection centers etc.) that are linked to both a relevant unique identifier and spatial co-ordinates could resolve this issue. They would need to be accessible and archived appropriately to facilitate later use.

The ability to accurately count and identify individual sheep (Challenge 3) should have improved with the introduction of new technologies, such as EID, and regulations requiring their use. This is the case; however, these methods have raised new issues. These include additional costs to production, mis-reads due to loss or failure over time (32–34), as well as variable attitudes to their adoption (35), while not entirely eliminating the potential for human error. In this study, the presence of dual recording of one “move” at two CCPs raised the potential to over-estimate the number of sheep moved to slaughter. When the additional market-to-slaughter records were removed, the apparent slaughter statistics derived from the movement data more closely matched those from national slaughter statistics. The use of the individual animal-level read records to facilitate the identification and removal of these market moves was not possible in 2006 (20). It now enables an improved indication of the direct spatial origin of the slaughter population.

The fourth challenge is that it is not currently possible to determine whether a batch movement contains lambs, adult sheep, or a mix of both. This is a major limitation of the sheep movement data not only for this study but for many surveillance purposes, Age-stratification of the data is important for sheep, as very often the epidemiology of the disease, condition, or subject under investigation is associated with age-group e.g., pneumonia in lambs, or ovine pulmonary adenocarcinoma in older sheep. The majority of moves to GB slaughterhouses will consist of lambs (29). However, within the industry sector it is well-known that, in addition to economic influences, there are both seasonal influences on the lamb throughput (29) and spatial influences on the preferred slaughter destination of ewes and rams. A substantial portion of the Scottish lamb population is known to go south over the border for slaughter, while the majority of Scottish cull ewes go direct to slaughter south of the border (36). It is highly likely that at least one of the high ranked non-Scottish slaughterhouses for receipt of the SSSP in Figure 4 is a major recipient of cull ewes, but this cannot be confirmed, or accounted for, based on the current sheep movement data. Historically, additional information on individual slaughterhouse throughput in terms of carcass weights could be used to approximately identify appropriate age-groups (20, 37). Such data are no longer easily accessible. Based on the derogation for those lambs that go direct to slaughter, from their holding of birth, at <12 months of age it should be possible to deduce their age from the use of a single flock-mark only slaughter tag (https://www.gov.scot/publications/sheep-goat-identification-traceability-guidance-keepers-scotland/pages/7/). However, it would still omit identification of the subset of the lamb population that do not go direct to slaughter from their holding of birth, such as store lambs and those lambs intended as replacement breeding stock that do not make selection for the breeding flock. A possible alternative might be to record whether the batch consists of lambs <12 months; sheep over 12 months, or a mix of both at a batch-level. Either of these approaches would be a substantial improvement. These data are rich and much more could be done with them, if sheep could be age-differentiated, as was the case with the Australian sheep movement data (16).

The fifth challenge of being able to identify only the direct move reduces the accuracy of the derived slaughterhouse catchment areas. As mentioned earlier, there will be a subset of the lamb population that does not move direct to slaughter, while adult sheep may have been resident on a number of premises within their life-time. Such movements contribute to the spatial dynamics observed in the outputs of the within-country non-slaughter movement analysis. Unlike cattle, where the individual animal passport and tracing system facilitates the modeling of life-time pathways (38–40), the missing individual reads for some batch movements make this more difficult for sheep. Studies are ongoing within the EPIC programme to develop statistical methods to address this (https://www.epicscotland.org/our-people/stephen-catterall/). A further problem arises when there are man-drawn borders within the one landmass with different recording systems on each side. Without access to the data from the other side, analyses of movements are compromised. Here, this occurs due to devolved responsibilities. In this study, some of the overall Scottish sheep out moves may have been to markets across the border, from whence they may have gone to other holdings, returned to slaughter in Scotland in population 4, or gone on directly to slaughter for which they would currently not be included in the SSSP as they did not go direct from a Scottish premises to a GB slaughter house. The extent of these effects cannot be quantified.

A key outcome of this study is the ability to use sheep movement data to start to identify and characterize potential sources of bias in slaughter-house based surveillance. The outputs indicate that any sheep slaughterhouse-based surveillance, conducted in Scottish slaughterhouses, that aims to be representative of the Scottish sheep slaughter population has the potential to produce biased estimates. If the disease, condition, or subject under investigation is systematically different between those holdings that only supply slaughter sheep to GB slaughterhouses out-with Scotland (SOSP) and those that contribute to the SISP, such a bias will exist. Given the spatial differences between the SISP and the SOSP holdings biased estimates are likely, particularly if management systems, breed, flock size, livestock or holding density, or weather conditions are associated with the disease, condition, or subject under investigation.

The AMR survey was a good exemplar to demonstrate how these analyses of sheep movement data can be applied to slaughterhouse-based survey design and data interpretation. For convenience sampling, the choice of the Scottish slaughterhouse with the largest ovine throughput is logical. However, by using the movement data, it has been demonstrated that there is potential for bias in the outcomes of the AST, if there is a systematic relationship between slaughter sheep from these areas and their carriage of resistant fecal *E. coli* i.e., in the frequency of occurrence of AMR to any of the active substances that were tested for. This could arise at three levels: firstly, due to the reduced coverage of the catchment area of this slaughterhouse; secondly, due to the difference between the catchment area and the slaughtered in Scotland sheep population (SISP), and thirdly because of the difference between the SISP and SSSP. The fact that there was no sampling in the AMR survey in the second quarter is probably of the least concern. This is the quarter with the least movement to slaughter; however, it could be one where older lambs, more likely to have been treated with antimicrobials may be sent to slaughter. Bias will occur if there are systematic differences between the levels of AMR in those sheep sub-populations from the areas not sampled and those sampled. If the areas missed were areas of more intensive, lowland sheep production, then it might be argued that the likelihood of AMR could be higher, due to increased antimicrobial usage during the lambing and/or fattening period, than in more extensive areas. This would lead to an underestimate of prevalence of AMR. And, vice versa, if the areas not sampled are those of extensive hill flocks with lower antimicrobial usage. The potential contribution to the existence of AMR in sheep fecal organisms from other spatially related factors such as the environment (41) may also be relevant.

An additional outcome is the potential to use these movement data to improve the sampling strategy so that it is more representative of a defined population of interest. For example, by sampling in the other top three ranked Scottish slaughterhouses in addition to slaughterhouse A, some of the gaps in coverage of the SISP population could be mitigated. Amendments to the sampling strategies could be explored. For example, to determine whether it would be of sufficient scientific advantage to sample either in the Island slaughterhouses, or to weight sampling in the south, and how that would be balanced by the feasibility, cost and resources required. Alternatively, if the sampling strategy is kept the same year-on-year to try to achieve a comparable sample, the analyses could be repeated to assess if this was indeed so.

With minimal additional resources, the applications that have been developed in this study could be used for other surveillance purposes. The movement matrices presented provide a visual representation of the dynamics of the within-Scotland non-slaughter sheep movements that can easily be replicated on updated movement data for any defined time period, with any defined scale for the categorization of number of movements. If such analyses were to be run on a routine basis, they could provide background information that could facilitate situational awareness (42) for those involved in animal health surveillance activities in Scotland. If run on a specified time period appropriate to the particular disease, or hazard, at notification of an outbreak, or incident, they might provide an early indication of where resources may need to be targeted, or mobilized, while more specific tracing programmes and models are being run. This may not be absolutely necessary when distances are small, areas easily accessed and sufficient field-based resources exist. However, it may be of use in converse situations.

Assessments of the quality and utility of movement data are rarely published in the scientific literature. There is a dearth of explicit studies (15), with only brief mentions of data quality, or challenges, within descriptive analyses (43, 44) that in more complex analyses are often relegated to single phrases such as “…numerous inconsistencies in the available data prevent…” (13), or “…anomalies in movement records and complexity of data processing…” (18). Often such assessments are relegated to reports to data providers and vanish into the depths “grey” literature (20, 45). Similarly, publications of slaughterhoused based initiatives only occasionally seek to characterize any potential bias. Mostly there is either a reliance on a sampling methodology that is structured to be representative [e.g., (46, 47)], or a brief mention that a potential for bias does, or doesn't, exist [e.g., (48)], with limited attempts for more in depth assessments, such as that in (49) or the more comprehensive investigation of active sheep TSE surveillance (19). If animal health surveillance is to develop, then these types of assessments are important foundations that need to be laid, regularly evaluated and easily available to users of the data.

In conclusion, firstly, the sheep movement data, currently collated and held in the EPIC data repository from ScotEID, can be used to inform the design and interpretation of Scottish sheep slaughterhouse-based surveillance activities. Secondly, data derived from sheep at Scottish slaughterhouses does have the potential to be biased in relation to spatially related attributes. Thirdly, the sheep movement data can be used to start to identify and characterize these potential biases, as well as to illustrate the dynamic nature of the movement of the sheep population within Scotland.

The regulatory requirement for the use of EID for individual sheep identification and centralized movement recording has improved the quantity of data available for use. Further steps are required to improve the quality of these Scottish data and to further develop this type of resource, so that their value can be fully realized and they can contribute to operational animal health surveillance. The most important “next step” with these Scottish data is to resolve the lack of information on the age of sheep being moved.

Estimates from any existing ovine slaughterhouse data, such as the AMR survey, need to be interpreted within the context of the identified potential for bias. The type of characterization presented here could—and should—be utilised at the design stage of future slaughter-house based studies. This need not be confined to sheep. The relatively simple, but computationally intense, analytical principles could be extended to other species and to livestock movement databases in other countries. However, sufficient attention will need to be paid to identification of the individual idiosyncrasies inherent in each database.

For other surveillance purposes, discussions with relevant stakeholders are required to determine what types of reports would be useful both for situational awareness, as well as for early resource allocation in outbreak situations. More generally, if the use of movement data for surveillance purposes is to be optimized, robust analyses like those presented here can only be achieved by interdisciplinary teams that are appropriately resourced, with access to multiple data sources facilitated by initiatives such as the EPIC data repository. This way informed exploratory analyses can be undertaken, development needs identified and animal health surveillance activities improved, for the mutual benefit of stakeholders, be they policy-makers, data-providers, surveillance professionals, the livestock industry, or the wider public.

## Data Availability Statement

Data have been obtained from a third party. The data analyzed in this study were obtained from the EPIC Data Repository and from SRUC Veterinary Services. Requests to access these datasets should be directed to the corresponding author for forwarding to the appropriate contact.

## Author Contributions

ST led the study (concept, design, and direction), interpreted the outputs (epidemiological and sheep sector knowledge) and jointly drafted the manuscript, with JS. JS devised and performed the data analysis, generated bespoke C++ applications for data handling and jointly drafted the manuscript with ST and produced figures. She is a member of the EPIC Data Team. JE provided statistical input and drafted the statistical methods. GF led the AMR survey (concept, microbiology, and data acquisition). AR provided advice regarding data handling and extraction, plus contributed to the development of the bespoke C++ applications. He leads the EPIC Data Team and led development of the EPIC Data Repository. JE, GF, and AR reviewed and commented on the manuscript. GG acquired the funding for and initiated the development of the EPIC Data Repository. He reviewed the manuscript. All authors read and approved the submitted version.

## Conflict of Interest

The authors declare that the research was conducted in the absence of any commercial or financial relationships that could be construed as a potential conflict of interest.
