# Supplementary figures and images for: The Use of Sheep Movement Data to Inform Design and Interpretation of Slaughterhouse-Based Surveillance Activities
Source: Front Vet Sci. 2020 Apr 24;7:205. doi: 10.3389/fvets.2020.00205 (PMC7193055; doi:10.3389/fvets.2020.00205)

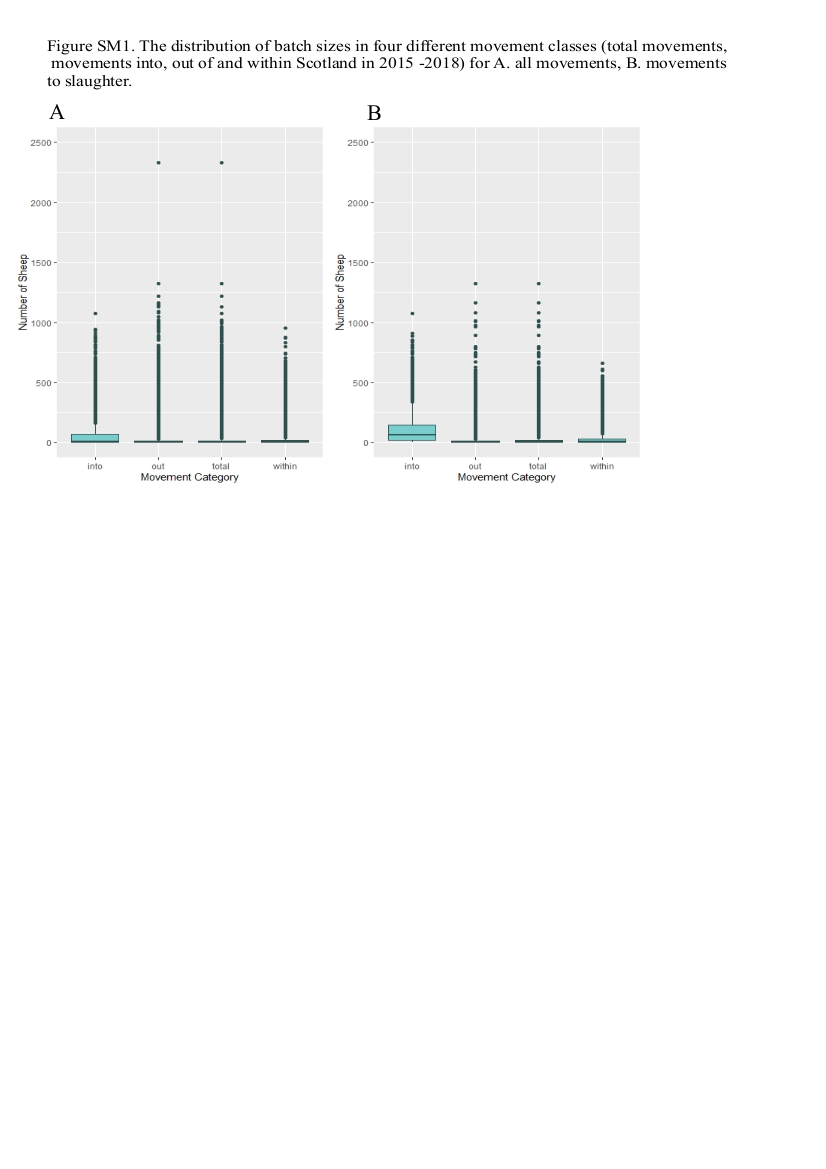

Supplement: Supplementary file 1 [file Image_1.TIF]

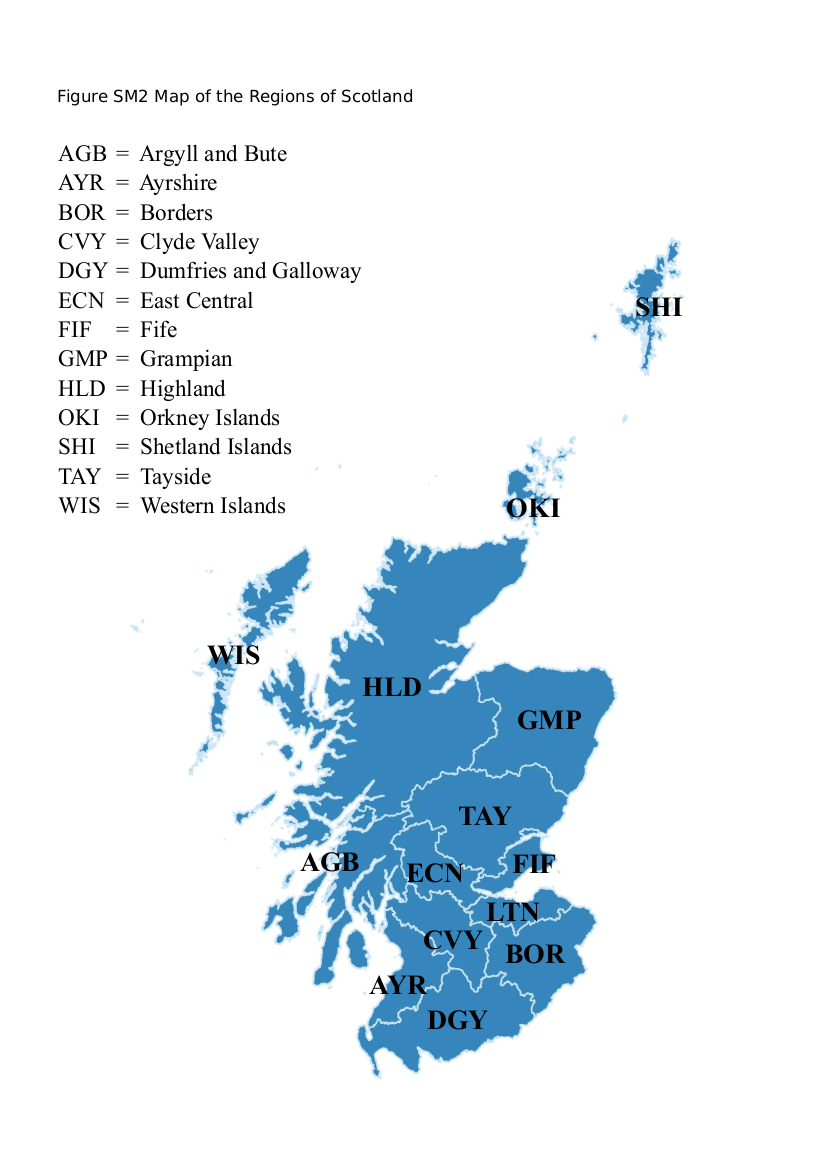

Supplement: Supplementary file 2 [file Image_2.TIF]

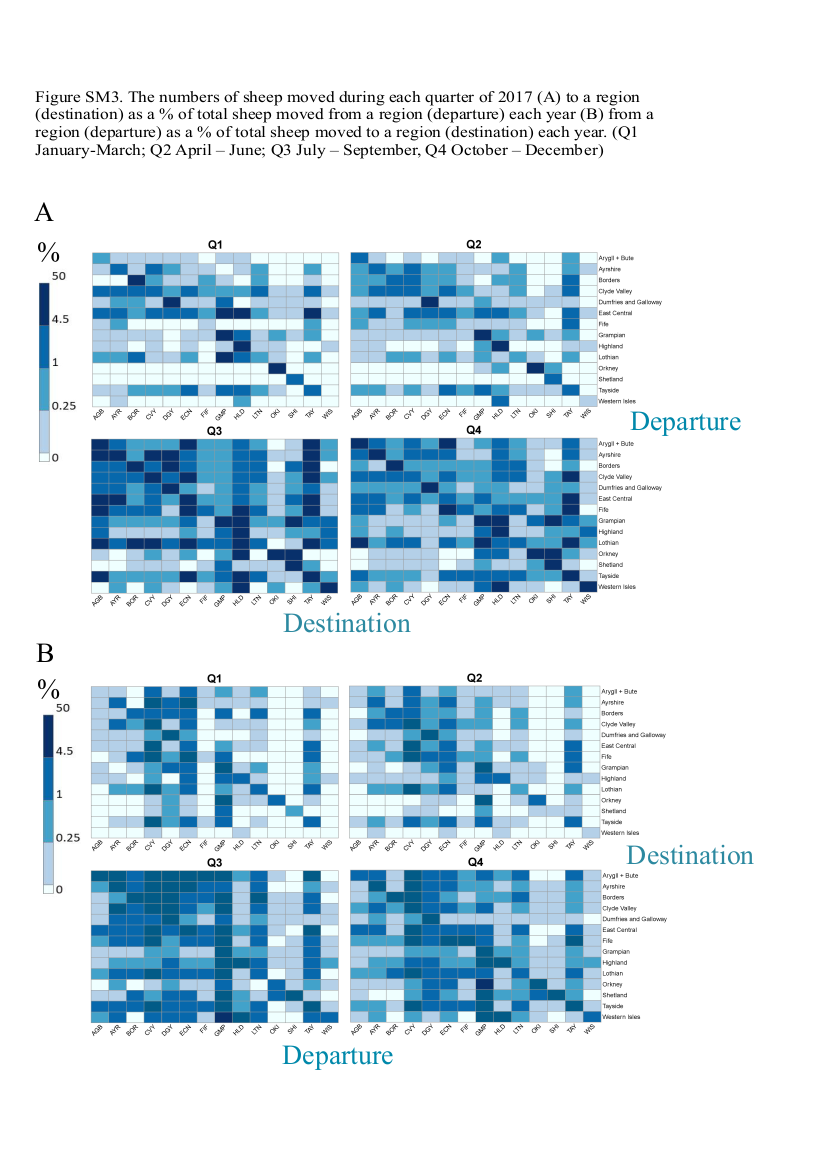

Supplement: Supplementary file 3 [file Image_3.TIF]
